# Supplementary material for: Acibenzolar-S-Methyl Activates Stomatal-Based Defense Systemically in Japanese Radish
Source: Front Plant Sci. 2020 Oct 30;11:565745. doi: 10.3389/fpls.2020.565745 (PMC7661486; doi:10.3389/fpls.2020.565745)
Supplement: Supplementary file 1 [file Data_Sheet_1.pdf]

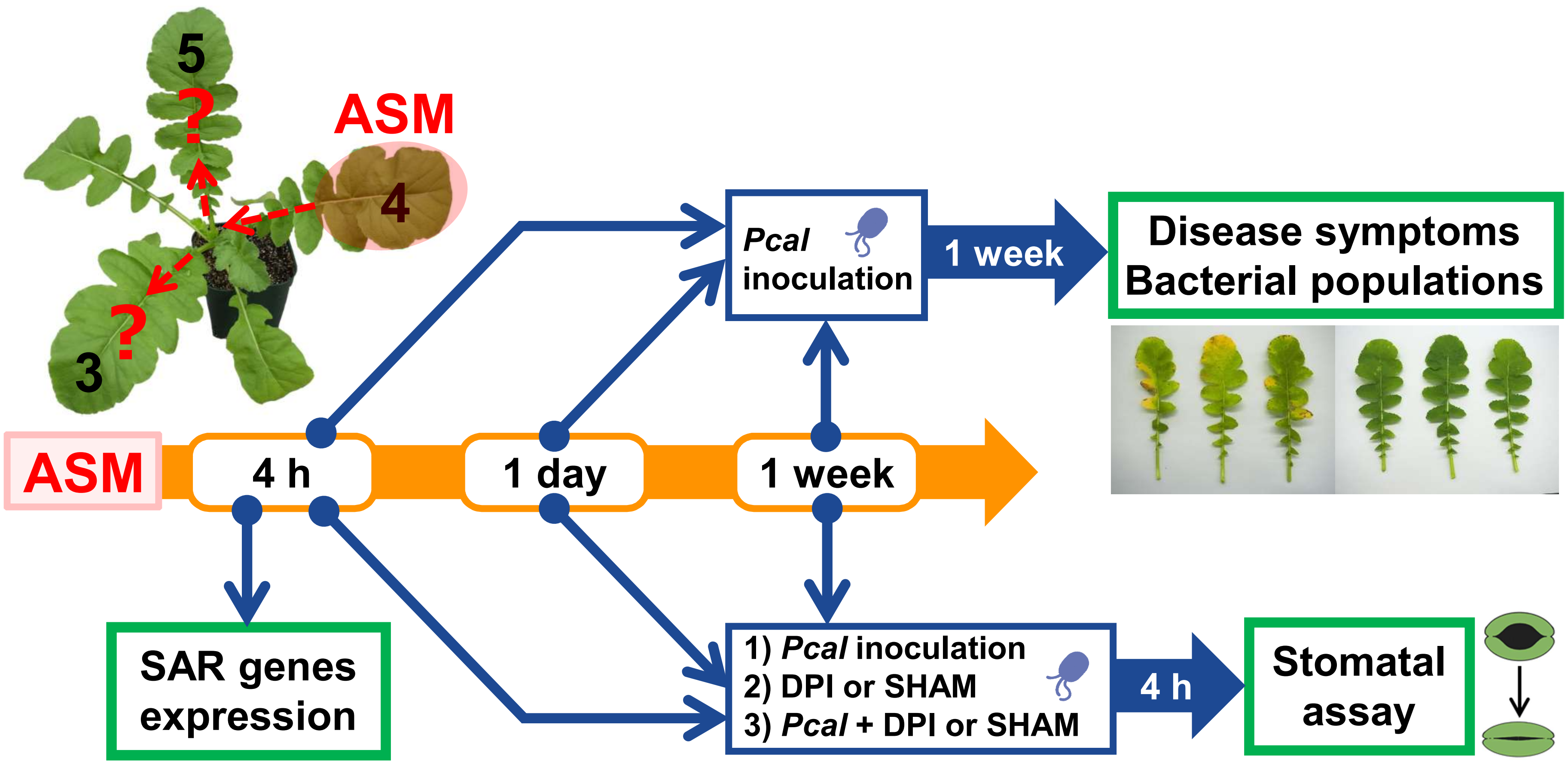

Supplementary Figure 1. The workflow of this study. Japanese radish plants were inoculated with *Pcal* 4 h, 1 d, and 1 w after ASM dip-treatment on only the fourth leaf. Disease symptoms (area and bacterial population) were assessed at 1-week post-inoculation (wpi). For stomatal assay, plants were 1) inoculated with *Pcal*, 2) treated with DPI and SHAM, and 3) inoculated with *Pcal* and treated with DPI and SHAM, 4 h, 1 d, and 1 w after ASM dip-treatment on only fourth leaves. For SAR genes expression profiles, plants were dip-treated with ASM on fourth leaves. Total RNAs were extracted 4 h after dip-treatment with ASM on fourth leaves and then used for RT-qPCR.

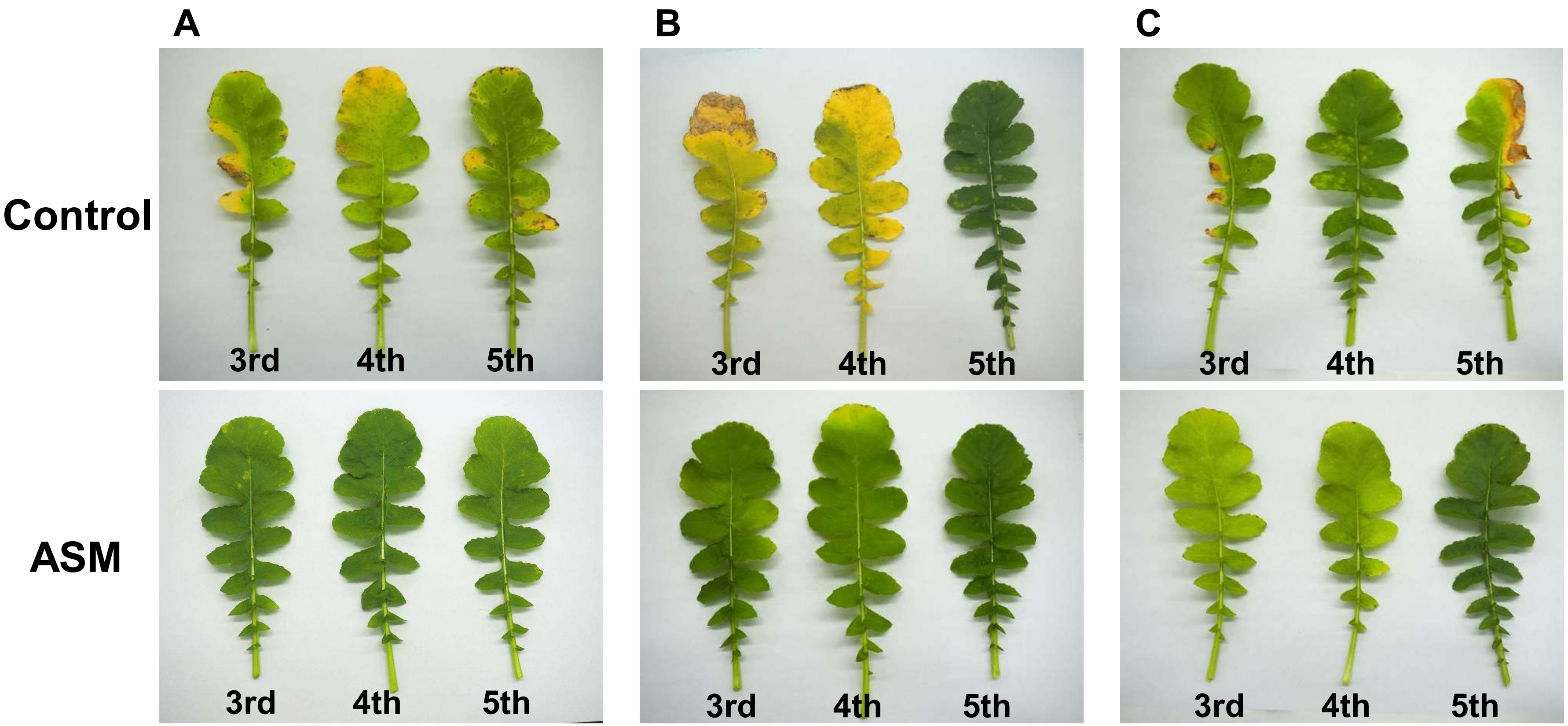

Supplementary Figure 2. Symptom development of Japanese radish inoculated with *Pcal* after the dip-treatment with ASM on fourth leaves. Greenhouse grown Japanese radish plants were spray-inoculated with *Pcal* (5×10^7 CFU/ml) 4 h **(A)**, 1 d **(B)**, and 1 w **(C)** after ASM dip-treatment (100 ppm) on fourth leaves. Disease symptoms were observed 7 days post-inoculation.

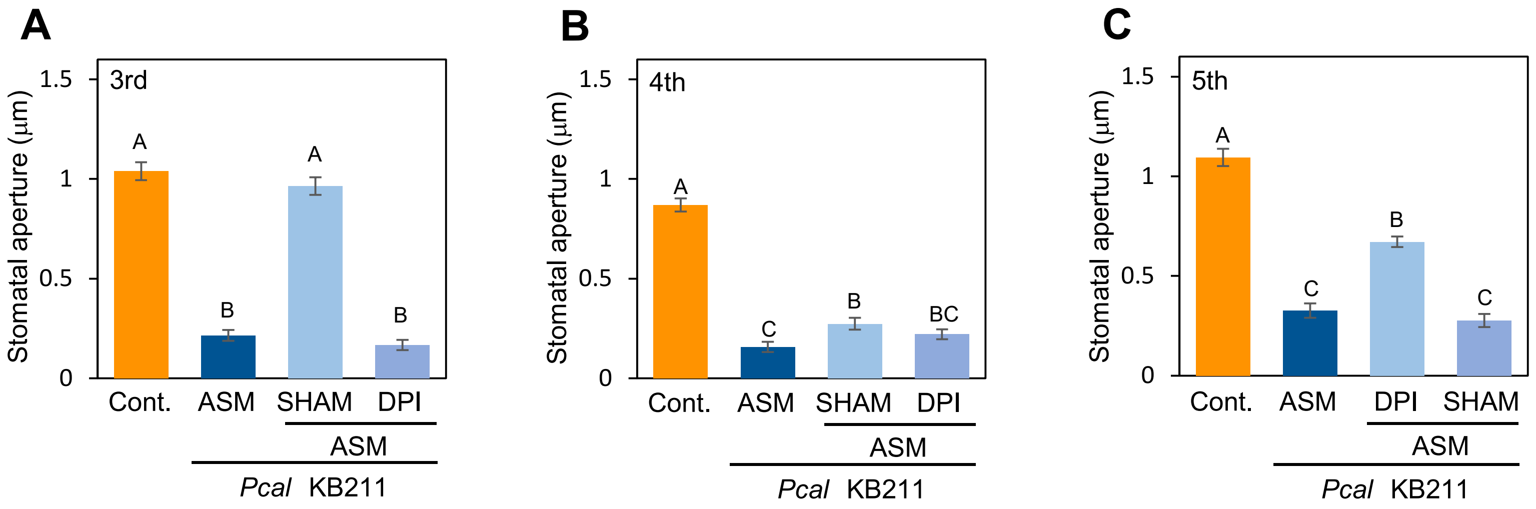

Supplementary Figure 3. Stomatal aperture width (µm) in Japanese radish plants dip-inoculated with *Pcal* suspensions (1 × 10^8 CFU/ml) and treated with SHAM and DPI after ASM treatment. Japanese radish leaves were inoculated with *Pcal* and treated with SHAM (1mM) and DPI (10 µM) after ASM dip-treatment (100 ppm) on forth leaves. Stomatal aperture width (µm) was measured on third **(A)**, fourth **(B)**, and fifth **(C)** leaves using a Nikon optical microscope. In all bar graphs, vertical bars indicate the standard error for three biological replicates. Significant differences ($p < 0.05$) are indicated by different letters based on a Tukey's honestly significant difference (HSD) test.

Supplementary Table 1. *p* values of the two-way ANOVA in Figure 3

Figure	Panel	Interaction	ASM	*Pcal*
3	A	7.876E-09	< 2.2e-16	0.06649
	B	< 2.2e-16	2.33E-12	8.78E-06
	C	5.19E-12	< 2.2e-16	0.006875
	D	4.09E-07	3.75E-16	0.007612
	E	0.21287	< 2e-16	0.03008
	F	0.045658	< 2.2e-16	0.001704
	G	0.00003639	< 2.2e-16	0.5139
	H	0.9113	< 2e-16	0.9626
	I	0.000005886	< 2.2e-16	0.004769
